# The Influence of Gender Roles on Eating Attitudes: A Study Among Female College Students Abstract

**DOI:** 10.1192/j.eurpsy.2024.1670

**Published:** 2024-08-27

**Authors:** B. Ozel, Y. Hosgören Alıcı, O. M. Kocak, S. Ceran

**Affiliations:** Department of Psychiatry, Başkent University, Ankara, Türkiye

## Abstract

**Introduction:**

Eating disorders (ED) are serious mental and physical illnesses that involve complex and damaging relationships with eating, exercise, and body image. They emerge due to a multifaceted interplay of factors, including familial predispositions, personality traits, and cultural influences. While societal beauty standards are recognized as significant risk factors, it is hypothesized that the roles and responsibilities associated with adult womanhood may also contribute to their development. In particular, the unique challenges faced by women, especially in developing countries like Turkey, may lead to discontent with traditional gender roles.

**Objectives:**

This study aims to explore the connection between eating disorders, female identity perceptions, body attitudes, expectations regarding women’s roles within families, and their potential association with body dysphoria. We investigate whether eating disorders are linked to a form of sexual dysphoria and body dysmorphia related to femininity rather than solely driven by societal beauty ideals.

**Methods:**

Data from 228 female college students, both undergraduate and graduate, were collected via online surveys. The survey instruments included a sociodemographic form, the Eating Attitude Test, the Gender Roles Attitude Scale, and the Multidimensional Body-Self Relations Questionnaire.

**Results:**

The average age of the participants was 24.41 (18-33) years. Regression analysis revealed that age (β=-0.155, p=0.015), the belief that physical appearance would be less important if they were male (β=0.292, p<0.001), and maternal criticism about weight (β=0.239, p<0.001) were influential factors in shaping eating attitudes. Surprisingly, no significant relationship was found between eating attitudes and traditional gender roles (β=0.072, p=0.246). However, we did establish a connection between aspiring to meet ideal thinness standards and perceiving women as disadvantaged in the workplace due to their traditional gender roles (t(226)=2.32, p=0.021), as well as with maternal criticism (t(225)=3.55, p<0.001).

**Conclusions:**

Our findings suggest that the absence of a direct link between eating attitudes and traditional gender roles may be attributed to an individual’s perception of their environment rather than their self-assessment of masculinity within an egalitarian context. Notably, maternal influences specifically their criticism regarding their daughters’ weight and the roles assigned to mothers significantly shape these perceptions and, consequently, eating behaviors, aligning with existing literature (Ferreira et al., 2021). This underscores the need to consider eating disorders within a broader biopsychosocial framework, encompassing attitudes toward the world and one’s role within it.

**Disclosure of Interest:**

None Declared

